# Poisoning by Chloral

**Published:** 1876-01

**Authors:** 


Poisoning by Chloral.—An interesting case of poisoning by Chloral hydrate is reported in the *Centralblatt f. d. Med. Wissench.* of April 3. A man who had taken twenty-four grammes (about 370 grains) of chloral hydrate, was found half an hour after in deep sleep, no more dangerous manifestions of its effects having yet developed themselves. About half an hour later, however, he began to suffer from interrupted respiration, the heart remaining normally active. Subsequently the heart's impulse became dangerously feeble, so that the pulse could only be felt in the carotid, while the face became deathly pale. The pupils were greatly contracted, and the temperature sank to 91.94 Fah. Artificial respiration by means of passive motions and faradization, being followed by no improvement, 0.003 gramme of strychnia was injected subcutaneously. Muscular spasm set in immediately, rapidly followed by trismus, the heart's impulse became again perceptible, the pupils enlarged, and the temperature rose to 91.94 Fah. Dangerous symptoms manifesting themselves again shortly after, a second injection of 0.002 grammes of strychnia was administered, the effects of which displayed themselves as before; the heart's action increased in power, and the temperature rose to the normal; respiration had, however, to be excited for eight hours longer by means of the induced current. Thirty-two hours after the poisoning he awoke fresh and free from all effects, having, on several occasions, been easily awakened from his sleep. The trismus and tetanic contraction of the muscles of the upper extremities persisted for fourteen hours after the second injection of strychnia. No gastritis followed this enormous dose of chloral, the probable reason being that the stomach was full at the time it was taken.—*New Remedies.*

